# Malware propagation model for cluster-based wireless sensor networks using epidemiological theory

**DOI:** 10.7717/peerj-cs.728

**Published:** 2021-09-15

**Authors:** Xuejin Zhu, Jie Huang

**Affiliations:** 1 School of Cyber Science and Engineering, Southeast University, Nanjing, Jiangsu, China; 2 Purple Mountain Laboratories, Nanjing, Jiangsu, China

**Keywords:** Malware propagation, Cluster-based WSNs, Equilibrium point, Game theory, Basic reproductive number

## Abstract

Due to limited resources, wireless sensor network (WSN) nodes generally possess weak defense capabilities and are often the target of malware attacks. Attackers can capture or infect specific sensor nodes and propagate malware to other sensor nodes in WSNs through node communication. This can eventually infect an entire network system and even cause paralysis. Based on epidemiological theory, the present study proposes a malware propagation model suitable for cluster-based WSNs to analyze the propagation dynamic of malware. The model focuses on the data-transmission characteristics between different nodes in a cluster-based network and considers the actual application parameters of WSNs, such as node communication radius, node distributed density, and node death rate. In addition, an attack and defense game between malware and defending systems is also established, and the infection and recovery rates of malware propagation under the mixed strategy Nash equilibrium condition are given. In particular, the basic reproductive number, equilibrium point, and stability of the model are derived. These studies revealed that a basic reproductive number of less than 1 leads to eventual disappearance of malware, which provides significant insight into the design of defense strategies against malware threats. Numerical experiments were conducted to validate the theory proposed, and the influence of WSN parameters on malware propagation was examined.

## Introduction

Wireless sensor networks (WSNs) are multi-hop self-organizing network systems formed by large-scale sensor nodes communicating with each other deployed in the monitoring area. With the rapid development of the Internet of Things and sensor technology, WSNs are widely used in various civilian and military fields, such as in smart cities, environmental monitoring, and data collection (Ahmad et al., 2017; García et al., 2020; Khalifeh et al., 2021; Cai & Mirrabbasi, 2021; Lazarescu & Poolad, 2021). However, due to the limited energy and computing resources of sensor nodes, it is difficult to construct a complex security protection system (Farjamnia, Gasimov & Kazimov, 2019). To reduce costs, manufacturers of sensor-node devices could neglect security. At the same time, sensor nodes are usually deployed in an open environment, which provides convenience for attackers (Liu et al., 2020). WSNs face many security threats, including denial-of-service (DoS) attacks, node capture, malware infection, and others (Souissi, Ben Azzouna & Ben Said, 2019). WSN security issues have attracted wide attention in academic circles and industrial circles.

Malware is a computer program that threatens or harms a network or system. The most common malware includes viruses, worms and Trojan horses, among others. In a WSN application, data are transmitted between adjacent nodes through wireless links, which creates favorable conditions for the propagation of malware. Malware may cause node failure, data leakage, DoS, and other failures., and can infect surrounding nodes through wireless transmission (Ojha et al., 2021). An attacker can target certain nodes, inject malware, and use the propagation mechanism to propagate the malware to the entire network, resulting in damage to the entire network system. However, the WSN system generally deploys protective measures against malware propagation that can detect data sent by system nodes and repair the nodes infected with the malware. However, implementation of these protective measures will predominately occupy limited communication channel resources and consume node power, which will increase data-transmission delays and shorten the lifecycle of network nodes. Therefore, it is necessary to choose an appropriate defense strategy to suppress the propagation of malware and minimize the loss of the entire system (Zhou, Shen & Liu, 2020). Therefore, it is vital to study the principles and mechanisms of malware propagation under attack and defense scenarios in WSNs.

In traditional Internet scenarios, epidemiological models have been widely used to study malware propagation, and a large number of computer malware propagation models have been proposed (Chen & Ji, 2005; Wang et al., 2014). However, because WSNs have the unique characteristics of limited node energy, node communication radius, and high node density, the mechanism of WSN Internet malware propagation clearly differs from traditional Internet scenarios. Therefore, the computer malware propagation model cannot be directly applied to malware propagation in WSNs.

In this study, the malware propagation model for cluster-based WSN network topology is evaluated based on game theory and epidemiology. Its primary contributions are as follows:

(1) An attack-defense game is proposed between malware and a WSN defend system. The mixed strategy Nash equilibrium of the model is obtained, and the infection rate of malware and the recovery rate of the system is calculated based on the Nash equilibrium solution of both parties in the game.

(2) A malware propagation mathematical model for cluster-based WSNs (based on the classic SIR model) is proposed under the attack-defense game. The model considers the data-transmission characteristics of cluster head nodes and common nodes, as well as the actual application scenarios and characteristics of WSNs, including node communication radius, node density, and node death.

(3) The basic reproductive number *R*_0_ and equilibrium point of the model are deduced, and the stability of the equilibrium point proved. When *R*_0_ < 1, WSN malware eventually disappears; otherwise, WSN malware will exist consistently.

(4) Numerical simulations are proposed for the proposed model; the experimental results support the correctness and effectiveness of the proposed model and elucidate the relationship between malware propagation and WSN parameters.

The remainder of the paper is organized as follows. In ‘Related Work’, related work on malware propagation is discussed. In ‘Proposed Model’, a novel malware propagation model for cluster-based WSNs under an attack-defense game is introduced. In ‘Existence and Stability of Equilibrium’, the equilibrium points and stability of the model system are deduced. The simulation and numerical analysis results for the malware propagation model are presented in ‘Simulation and Numerical Analysis’. Conclusions are drawn in ‘Conclusion’.

## Related Work

Research toward exploring the malware propagation behavior of WSNs has resulted in several achievements. A robust survey has summarized malware propagation models in networks (Queiruga-Dios et al., 2017). In a study evaluating the propagation dynamics of worms in time and space in WSNs, Khayam & Radha (2005) considered the physical, MAC, and network layers of actual sensor networks according to the topology characteristics of WSNs, and proposed the topologically aware worm propagation model (TWPM). Shen et al. (2016) proposed a malware propagation model based on the epidemiology theory, and solved the problem of how to evaluate the reliability of sensor nodes in the case of malware propagation, so as to ensure efficient, continuous, and reliable transmission of sensory data from the node to the sink. Mishra & Keshri (2013) proposed an infectious disease model that includes a vaccination room. Their model not only reflects the temporal and spatial dynamics of the worm propagation process, but also performs mathematical analysis and numerical simulation on the worm propagation process. Nwokoye & Umeh (2018) developed the analytic-agent cyber dynamical systems analysis and design method (*A*^2^*CDSADM*) by combining the prevalent analytical and agent methods. This method can not only reflect the time dynamics of malware spreading, but can also observe the spatial dynamic changes of sensor nodes of different groups. Qiao et al. (2014) found that WSNs have the characteristics of small-world networks. They studied the epidemics in the small world of tree-based networks and calculated the epidemic threshold for epidemic outbreaks.

Many studies based on classic SIR epidemiology have also been undertaken. Wang & Li (2009) considered the energy of the sensor node to be limited; thus, based on the SIR model, the node death state was introduced to obtain a new model, *i.e.,* iSIRS. The authors further proposed the EiSIRS virus propagation model (Wang, Li & Li, 2010), which describes the process of worm propagation in WSNs with sleep mechanisms. Their experimental results demonstrate that the remaining energy of nodes and the sleep scheduling mechanism are effective against worms. The propagation of viruses in WSNs has a certain impact. Liping et al. (2015) proposed an improved SIRS worm propagation model that considers the communication radius and distribution density of WSN nodes, determines the model’s equilibrium and basic reproductive number, and obtains large-scale worm propagation conditions. Zhu, Zhao & Wang (2015) proposed a SIRS malware propagation model with a state-feedback controller. Through the analysis of model stability and Hopf bifurcation, the state-feedback method was successful for unstable stable states and periodic oscillation control. Tang et al. proposed an SI model based on node dormancy maintenance that provides a repair function when an infected node enters the dormant state (Tang & Mark, 2009; Tang, 2011). The improved SI model can effectively prevent virus propagation in the network without adding any additional hardware workload or computational overhead.

The attack and defense parties of malware in WSNs can be regarded as having a game relationship. Therefore, game theory is also widely used in the security of WSNs, especially in malware-related fields. Abdalzaher et al. (2016) proposed a node protection model based on a Stackelberg game, which can be adapted to two different malicious node attack scenarios. In the first scenario, the attacker selects a group of nodes for which the protection degree is lower than a certain threshold to attack. In the second scenario, the attacker’s goal is to defend the weakest node in the previous round of attack. Wang, Li & Dong (2018) proposed an improved two-dimensional (2D) cellular automata model and a multi-role evolutionary game model to describe the process of malware propagation. Based on the existing 2D cellular automata malware model, the epidemiological propagation mechanism is improved, and the dynamic equation of strategy evolution is given. Shen et al. (2017) proposed a non-cooperative non-zero-sum game to describe the interaction between heterogeneous WSNs (HWSNs) system and malware. The game model can predict infection behavior of malware. Further, the author has established a node reliability evaluation mechanism in the state of malware propagation, which can efficiently evaluate system availability and reliability. Shen et al. (2014) proposed a differential game model for maleware propagation in WSNs. In the process of the game between the system and the malware, the defense strategy can be changed dynamically, so that the total cost can be minimized. In addition, the author also considered the node sleep state in the process of propagation.

The current WSN malware propagation model is based on a flat network structure; in which all nodes in the network are equal and have completely consistent functional characteristics. However, the actual application scenarios of WSNs typically adopt a hierarchical network structure, and nodes are deployed in clusters. There are two types of nodes in WSNs, cluster head nodes and common nodes. This cluster-based network topology has many advantages over flat topology, such as easy expansion, convenient centralized management, low system construction cost, high network coverage, and reliability (Huang, Jie & Guizani, 2014). Because different network topologies adopt different data-transmission rules, the propagation mechanisms of malicious network software can vary substantially. Thus, previous studies cannot be applied to cluster-based hierarchical networks. In addition, existing epidemiological studies do not consider the strategies of infection rate and recovery rate, but only use a fixed parameter to express it, thus ignoring the impact of the attack and defense game process on the dynamics of malware propagation. In the actual propagation of WSN malware, both the cost and benefits of malware infection and system defense will be considered. For malware, if the expected benefit of launching an attack is greater than the cost, it will launch an infection attack. Otherwise, its malicious intentions will be hidden. For system defense, the data is received by the node detected only when the system benefit of detection and repair is greater than the cost of detection. In response to these problems, in this study the propagation processes of malware in WSNs are analyzed, and a more effective formal model established to accurately determine the propagation dynamics of malware with cluster-based hierarchical network structure.

## Proposed Model

**Figure 1:**
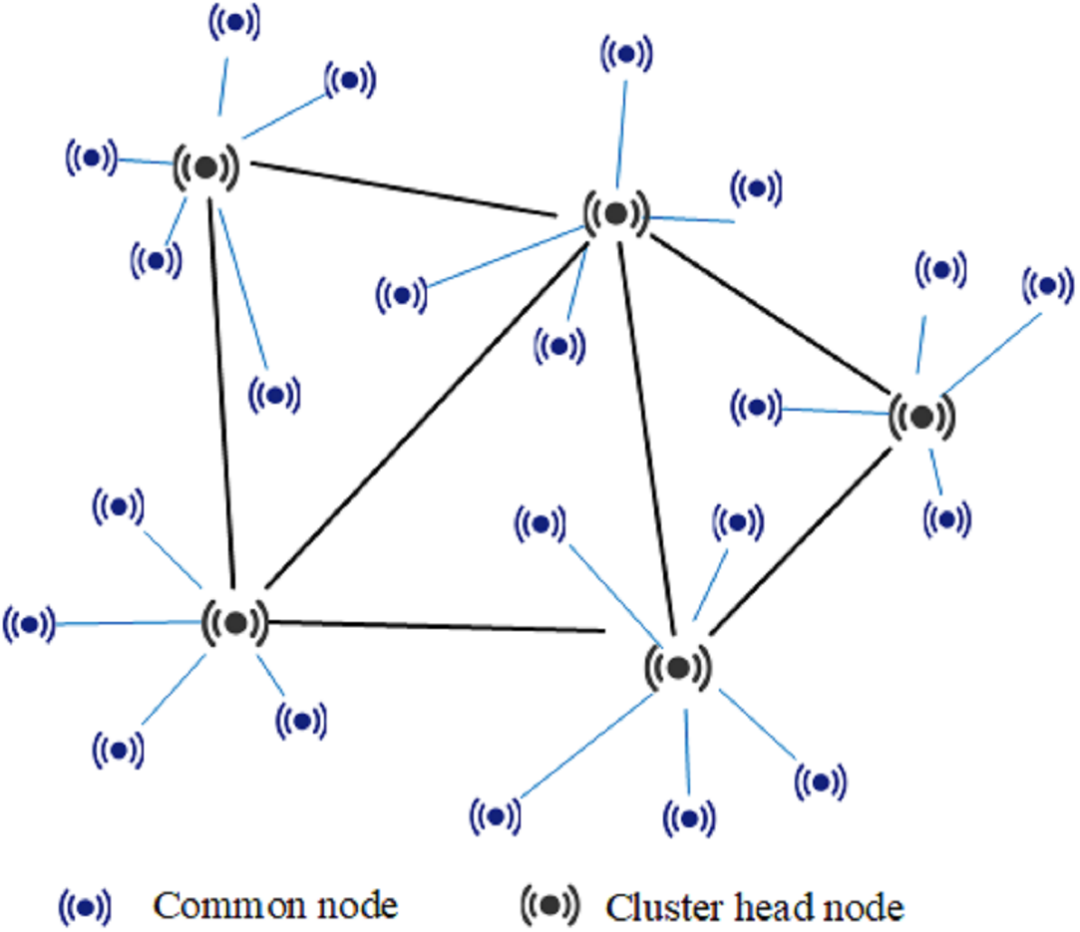
Topological structure of cluster-based WSNs.

The proposed identification method is presented in this section, but first the notations and their definitions that will be used in this paper are listed in Table 1. A cluster-based hierarchical WSN can be divided into cluster head nodes and common nodes according to the function of sensor nodes as shown in Fig. 1. Each cluster head node contains the same functional protocol, such as MAC address, routing, nodes management, or security protocol, while common sensor nodes usually do not have functions such as routing, management, and aggregation processing. The common nodes send collected data to the cluster head node. After data-fusion processing, the cluster head node transfers the data through a multi-hop routing and forwarding mechanism, and finally uploads it to the network base station. Therefore, common nodes can only communicate with the cluster head node of the cluster, but cluster head nodes can communicate with one another. It is assumed that sensor nodes are evenly distributed and deployed in a certain area, the communication radius of each sensor node is *r*, and the deployment density of the cluster head nodes is *σ*.

**Table 1:** Table of notations and definitions.

**Notation**	**Definition**
$\mathcal{M}/\mathcal{D}$	Malware/defend system
${C}_{\mathcal{M}}/{C}_{\mathcal{D}}$	Malware/defend system’s strategy space
${U}_{\mathcal{M}}/{U}_{\mathcal{D}}$	Malware/defend system’s utility
$\widetilde {S}(t)/S(t)$	Number of susceptible cluster head nodes/common nodes at time *t*
$\widetilde {I}(t)/I(t)$	Number of infected cluster head nodes/common nodes at time *t*
*R*(*t*)	Number of recovered nodes at time *t*
${\widetilde {S}}_{r}/{S}_{r}$	Number of effective contacts of cluster head nodes/common nodes
*N*_1_/*N*_2_	Number of cluster head nodes/common nodes in cluster-based WSNs
*β*	Infection rate of malware
*γ*	Recovery rate of infected nodes
*r*	Communication radius of sensor nodes
*b*	Birth rate of sensor nodes
*σ*	Density of cluster head nodes
*ɛ*	Cost of attack by infected node
*τ*	Cost of system repairing infected node
*ν*	Cost of security detection by susceptible nodes on received data packets
*e*	After infected node is restored, cost to malware or gain to system
*R*_0_	The basic reproductive number of malware propagation
*E*_0_	The malware-free equilibrium malware propagation
*E*_1_	The endemic equilibrium of malware propagation

According to the classic SIR epidemiological model, the network nodes can have the following three states.

• Susceptible (*S*): The susceptible nodes are in a healthy state but can be easily infected by malware. Before the system is attacked by malware, the susceptible state is the initial state of all sensor nodes. In the proposed model, nodes in the susceptible state are divided into susceptible cluster head nodes and susceptible common nodes.
• Infected (*I*): The infected nodes are in a state of being contaminated by malware and can send multiple copies of the malware to nearby nodes by data interaction, thereby infecting the nodes in state *S*. Similar to the susceptible nodes in the proposed model, the nodes in state *I* are divided into infected cluster head nodes and infected common nodes.
• Recovered (*R*): The recovered nodes are infected nodes that have been restored to a healthy state through security measures such as virus detection and vulnerability patching and will acquire immunity.

### Game between malware and defend system for WSNs

Before studying the propagation mechanism of WSNs malware, one must first consider the attack and defense strategy between malware and a defend system. For sensor nodes infected by malware, it can propagate to more surrounding nodes, which can make the network invalid in a large area. However, if the malware propagates too frequently, it is easy to be detected by susceptible nodes and repaired. At the same time, the energy of the infected node will be consumed every time the malware is propagated. Therefore, the problem for infected nodes is maximizing the propagation of malware while minimizing their own energy consumption. As for the defend system, the data received by the node can be detected for security. If abnormal malware packets are found, the data will be discarded, and the last hop node will be installed with patches and restored. However, these methods will inevitably bring interference to the normal operation of WSNs. For example, detecting data and installing security patches will consume energy and occupy bandwidth. Therefore, the defend system will also detect the received data with a certain probability, so as to maximize its own benefits. This shows that the attack and defense process between malware and defend systems is actually a game. Therefore, in this paper game theory is used to analyze the attack and defense strategies between them.

**Definition:** The strategic game between malware and a defend system in WSNs is composed of a ternary $\mathbb{G}=(J, \left\{ {C}_{i} \right\} , \left\{ {U}_{i} \right\} )$, where

• $J= \left\{ \mathcal{M},\mathcal{D} \right\} $ is the set of players in the game, where $\mathcal{M}$ and $\mathcal{D}$ represent the malware and the defend system, respectively.
• *C*_*i*_ is the strategy set of player *i*when *i* is the defend system; ${C}_{\mathcal{D}}= \left\{ detecting,nodetecting \right\} $, when *i* is malware; ${C}_{\mathcal{M}}= \left\{ propagation,nopropagation \right\} $.
• *U*_*i*_ is the utility obtained by the player *i* during the game, and its value is determined by the strategies adopted by both sides of the game.

According to the attack and defense game in Definition 1, the payoff matrix of the game can be obtained, as shown in Table 2. If the susceptible node performs security detection on the received data packet, it must consume energy and may block the channel, and the resulting system cost is recorded as *ν*. The cost of malware caused by the energy consumption of the infected node sending the data packet containing the malicious software to the next hop node is recorded as *ɛ*. After the susceptible node is detected, if malware is found, the system will repair the last hop node, which will consume energy and bandwidth, and set the total cost as *τ*. After the repair is successful, the utility to the system and the cost to the malware are both *e*. When the malware initiates an attack and the system initiates detection, the overall utility of the system is −*ν* − *τ* + *e*, and the utility of the infected node is −*e* − *ɛ*. When the system is not detected, the utility of the system and that of the malware are −*e* and *e* − *ɛ*, respectively. In addition, when the system initiates detection but no malware is found, the system’s utility will be −*ν*. In other cases, the utility of both parties is zero.

**Table 2:** Game payoff for defend system and malware.

**Defend system**	**Malware**	
	*Propagation*	*Non-propagation*
*Detection*	−*ν* − *τ* + *e*, −*e* − *ɛ*	−*ν*, 0
*Non-detection*	−*e*, *e* − *ɛ*	0, 0

The notion of mixed strategy Nash equilibrium (MNE) captures a steady state of a strategic game in which each player holds the correct expectation about other players’ behavior and acts rationally. The main feature of MNE is that the opponent adopts any pure strategy, and the player’s expected utility is the same. According to game theory, there must be MNE in the game of limited players, so one can solve the MNE of the game 𝔾. Assume that malware attacks and propagates with a probability of *p*, and does not attack with a probability of 1 − *p*. The system detects the received data with probability *q*, and does not detect with probability 1 − *q*. The utility maximization method to can be used to solve the MNE. According to the utility matrix, the expected utility of the system $E({U}_{\mathcal{D}})$ is

(1) \begin{eqnarray*}\begin{array}{@{}r@{}} \displaystyle E({U}_{\mathcal{D}}) & =pq \left( -\nu -\tau +e \right) +q \left( 1-p \right) \left( -\nu \right) & \\ \displaystyle & \hspace*{10.00002pt}+p \left( 1-q \right) \left( -e \right) \end{array}\end{eqnarray*}

Letting $ \frac{\partial E({U}_{\mathcal{D}})}{\partial q} =0$, one has

(2) \begin{eqnarray*}p= \frac{\nu }{2e-\tau } \end{eqnarray*}

Similarly, the expected utility of malware $E({U}_{\mathcal{M}})$ is

(3) \begin{eqnarray*}\begin{array}{@{}r@{}} \displaystyle E({U}_{\mathcal{M}}) & =pq \left( -e- \right) +p \left( 1-q \right) \left( e- \right) \end{array}\end{eqnarray*}

Letting $ \frac{\partial E({U}_{\mathcal{M}})}{\partial p} =0$, one has

(4) \begin{eqnarray*}q= \frac{e-}{2e} \end{eqnarray*}

When malware initiates an attack with probability $ \frac{\nu }{2e-\tau } $, the expected utility of the malware is the same regardless of whether the system is detected or not. Similarly, when the probability of system detection is $ \frac{e-}{2e} $, the expected utility is the same regardless of whether the malware initiates an attack. Therefore, (${p}^{\ast }= \frac{\nu }{2e-\tau } ,{q}^{\ast }= \frac{e-}{2e} $) is the MNE of the attack and defense game between malware and system. When both parties are rational, they will use this strategy to attack and defend. When the malware initiates an attack and the system does not detect it, the malware will spread successfully; when the malware initiates an attack and the system detects it, the system will find the malware and repair the data source node. Therefore, the malware infection rate and system recovery rate under MNE conditions can be obtained:

(5) \begin{eqnarray*}\beta ={p}^{\ast } \left( 1-{q}^{\ast } \right) = \frac{\nu \left( e+ \right) }{2e \left( 2e-\tau \right) } \end{eqnarray*}

(6) \begin{eqnarray*}\gamma ={p}^{\ast }{q}^{\ast }= \frac{\nu \left( e- \right) }{2e \left( 2e-\tau \right) } \end{eqnarray*}

### Malware propagation model

In this subsection, a malware propagation model is established under the condition of MNE. Let $\widetilde {S}(t)$, $\widetilde {I}(t)$, *S*(*t*), *I*(*t*), and *R*(*t*) denote the susceptible cluster head nodes, infected cluster head nodes, susceptible common nodes, infected common nodes, and number of recovered nodes at time *t*, respectively. According to the malware attack strategy under MNE, the infected nodes infect surrounding susceptible nodes with infection rate *β* that can communicate with each other. Moreover, according to the system defense strategy, the infected nodes recover at a rate of *γ*.

In a cluster-based hierarchical network, it is assumed that the number of cluster head nodes and common nodes are *N*_1_ and *N*_2_, respectively; that is, there are *N*_1_ clusters in the network, and each cluster contains *N*_2_/*N*_1_ common nodes with the node status conversions. Letting ${\widetilde {S}}_{r}(t)$ and *S*_*r*_(*t*) represent the effective contact number of the infected cluster head node against the susceptible cluster head node and the susceptible common node, respectively. The formula is expressed as follows:

(7) \begin{eqnarray*}{\widetilde {S}}_{r}(t)= \frac{\sigma \pi {r}^{2}}{{N}_{1}} \widetilde {S}(t)\end{eqnarray*}

(8) \begin{eqnarray*}{S}_{r}(t)= \frac{S(t)}{{N}_{1}} \end{eqnarray*}

**Figure 2:**
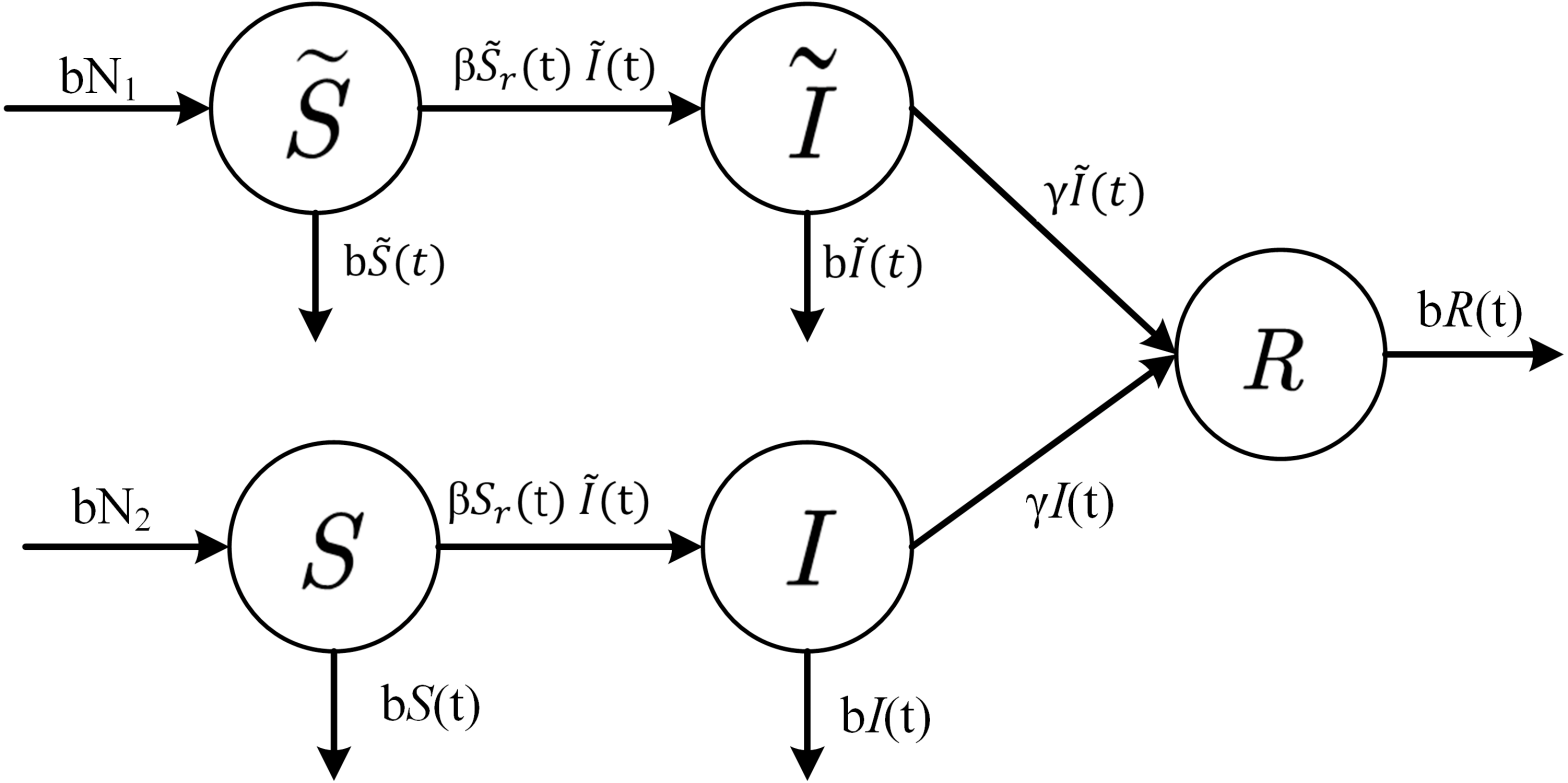
State-transition relationships of nodes in WSNs.

Each infected node can infect $\beta {\widetilde {S}}_{r}$ susceptible nodes per unit time. Therefore, the conversion rate of susceptible cluster head nodes to infected cluster head nodes is $\beta {\widetilde {S}}_{r}(t)\widetilde {I}(t)$ at time *t*. Since common nodes cannot communicate with each other but can only be infected by cluster head nodes, the infection rate of common nodes is also determined by $\widetilde {I}(t)$. The conversion rate from susceptible common node to infected common node is $\beta {S}_{r}(t)\widetilde {I}(t)$ at time *t*. At the same time, due to the existence of the defense system, the conversion rate of infected nodes *I* and $\widetilde {I}$ to immune group *R* is *γI*(*t*) and $\gamma \widetilde {I}(t)$, respectively. Considering that the node cannot continue to work due to physical device damage or exhaustion of battery power, the death and births rate of the node are both set to *b*, which ensures that the number of nodes in the network remains constant. In this way, we can obtain the state transition relationship between groups in the cluster network shown in Fig. 2.

In accordance with the rates of change between different states shown in Fig. 2, one can establish a mathematical model of a system of differential equations based on cluster-based WSNs for malware propagation:

(9) \begin{eqnarray*} \left\{ \begin{array}{@{}l@{}} \displaystyle \frac{d\widetilde {S}(t)}{dt} =b{N}_{1}-\beta \frac{\sigma \pi {r}^{2}}{{N}_{1}} \widetilde {S}(t)\widetilde {I}(t)-b\widetilde {S}(t)\\ \displaystyle \frac{d\widetilde {I}(t)}{dt} =\beta \frac{\sigma \pi {r}^{2}}{{N}_{1}} \widetilde {S}(t)\widetilde {I}(t)-\gamma \widetilde {I}(t)-b\widetilde {I}(t)\\ \displaystyle \frac{dS(t)}{dt} =b{N}_{2}-\beta \frac{S(t)}{{N}_{1}} {I}^{{^{\prime}}}(t)-bS(t)\\ \displaystyle \frac{dI(t)}{dt} =\beta \frac{S(t)}{{N}_{1}} {I}^{{^{\prime}}}(t)-\gamma I(t)-bI(t)\\ \displaystyle \frac{dR(t)}{dt} =\gamma \widetilde {I}(t)+\gamma I(t)-bR(t) \end{array} \right. \end{eqnarray*}

Since the total number of sensor nodes in the system is a fixed value (*N*_1_ + *N*_2_), one can obtain

(10) \begin{eqnarray*}R(t)={N}_{1}+{N}_{2}-\widetilde {S}(t)-S(t)-\widetilde {I}(t)-I(t)\end{eqnarray*}

Therefore, primarily the first four equations in system (9) are considered. Letting

(11) \begin{eqnarray*}{\alpha }_{1}=\beta \frac{\sigma \pi {r}^{2}}{{N}_{1}} \end{eqnarray*}

(12) \begin{eqnarray*}{\alpha }_{2}= \frac{\beta }{{N}_{1}} \end{eqnarray*}

The system model Eq. (9) can finally be simplified as

(13) \begin{eqnarray*} \left\{ \begin{array}{@{}l@{}} \displaystyle \frac{d\widetilde {S}}{dt} =b{N}_{1}-{\alpha }_{1}\widetilde {S}\widetilde {I}-b\widetilde {S}\hspace*{10.00002pt}\\ \displaystyle \frac{d\widetilde {I}}{dt} ={\alpha }_{1}\widetilde {S}\widetilde {I}-\gamma \widetilde {I}-b\widetilde {I}\hspace*{10.00002pt}\\ \displaystyle \frac{dS}{dt} =b{N}_{2}-{\alpha }_{2}S\widetilde {I}-bS\hspace*{10.00002pt}\\ \displaystyle \frac{dI}{dt} =\beta {\alpha }_{2}S\widetilde {I}-\gamma I-bI\hspace*{10.00002pt} \end{array} \right. \end{eqnarray*}

## Existence and Stability of Equilibrium

In this section equilibrium points of system Eq. (13) are derived and their stability proved. If an equilibrium point is globally stable, then the final state of the system is certain under any initial conditions. For equilibrium points of system Eq. (13), one has

(14) \begin{eqnarray*} \frac{d\widetilde {S}}{dt} =0, \frac{d\widetilde {I}}{dt} =0, \frac{dS}{dt} =0, \frac{dI}{dt} =0\end{eqnarray*}

Letting

(15) \begin{eqnarray*}{\alpha }_{1}\widetilde {S}\widetilde {I}-\gamma \widetilde {I}-b\widetilde {I}=0\end{eqnarray*}

one has (i) $\widetilde {I}=0$ or (ii) $\widetilde {S}= \left( \gamma +b \right) /{\alpha }_{1}$ and $\widetilde {I}\gt 0$. For the case of $\widetilde {I}=0$, one has malware-free equilibrium

(16) \begin{eqnarray*}{E}_{0}=({\widetilde {S}}_{0},{\widetilde {I}}_{0},{S}_{0},{I}_{0})=({N}_{1},0,{N}_{2},0)\end{eqnarray*}

For the case of $\widetilde {S}= \left( \gamma +b \right) /{\alpha }_{1}$ and $\widetilde {I}\gt 0$, one has endemic equilibrium:

(17) \begin{eqnarray*}{E}_{1}=({\widetilde {S}}_{1},{\widetilde {I}}_{1},{S}_{1},{I}_{1})\end{eqnarray*}

where

(18) \begin{eqnarray*}{\widetilde {S}}_{1}= \frac{\gamma +b}{{\alpha }_{1}} \end{eqnarray*}

(19) \begin{eqnarray*}{\widetilde {I}}_{1}= \frac{b{N}_{1}}{\gamma +b} - \frac{b}{{\alpha }_{1}} \end{eqnarray*}

(20) \begin{eqnarray*}{S}_{1}= \frac{b{N}_{2}}{{\alpha }_{2}{\widetilde {I}}_{1}+b} \end{eqnarray*}

(21) \begin{eqnarray*}{I}_{1}= \frac{b{N}_{2}}{(\gamma +b)(1+ \frac{b}{{\alpha }_{2}{\widetilde {I}}_{1}} )} \end{eqnarray*}

Letting

(22) \begin{eqnarray*}{R}_{0}= \frac{{N}_{1}{\alpha }_{1}}{\gamma +b} = \frac{\beta \sigma \pi {r}^{2}}{\gamma +b} \end{eqnarray*}

*R*_0_ is the basic reproductive number of system Eq. (13), and only if *R*_0_ > 1; thus, ${\widetilde {I}}_{1}\gt 0$, and the endemic equilibrium *E*_1_ is meaningful.

### Malware-free equilibrium and its stability

To investigate the local stability of the equilibrium points of the system Eq. (13), one must calculate the corresponding Jacobian matrix as

(23) \begin{eqnarray*}J= \left[ \begin{array}{@{}cccc@{}} \displaystyle -{\alpha }_{1}\widetilde {I}-b&\displaystyle -{\alpha }_{1}\widetilde {S}&\displaystyle 0&\displaystyle 0\\ \displaystyle {\alpha }_{1}\widetilde {I}&\displaystyle {\alpha }_{1}\widetilde {S}-\gamma -b&\displaystyle 0&\displaystyle 0\\ \displaystyle 0&\displaystyle -{\alpha }_{2}S&\displaystyle -{\alpha }_{2}\widetilde {I}-b&\displaystyle 0\\ \displaystyle 0&\displaystyle {\alpha }_{2}S&\displaystyle {\alpha }_{2}\widetilde {I}&\displaystyle -\gamma -b \end{array} \right] \end{eqnarray*}

**Lemma 1.** The malware-free equilibrium *E*_0_ of system Eq. (13) is locally asymptotically stable if *R*_0_ < 1 and unstable if *R*_0_ > 1.

**Proof.** Calculating the Jacobian matrix Eq. (25) at malware-free equilibrium, one obtains

(24) \begin{eqnarray*}J \left( {E}_{0} \right) = \left[ \begin{array}{@{}cccc@{}} \displaystyle -b&\displaystyle -{\alpha }_{1}{N}_{1}&\displaystyle 0&\displaystyle 0\\ \displaystyle {\alpha }_{1}\widetilde {I}&\displaystyle {\alpha }_{1}{N}_{1}-\gamma -b&\displaystyle 0&\displaystyle 0\\ \displaystyle 0&\displaystyle -{\alpha }_{2}{N}_{2}&\displaystyle -b&\displaystyle 0\\ \displaystyle 0&\displaystyle {\alpha }_{2}{N}_{2}&\displaystyle 0&\displaystyle -\gamma -b \end{array} \right] \end{eqnarray*}

To prove the stability at the point *E*_0_, one will find all the eigenvalues (*λ*) of the matrix Eq. (26). The eigenvalues are given as

(25) \begin{eqnarray*}\lambda =-b,\hspace*{10.00002pt}-\gamma -b,\hspace*{10.00002pt}{\alpha }_{1}{N}_{1}-\gamma -b\end{eqnarray*}

where −*b* is a double eigenvalue.

Hence, when *R*_0_ < 1, all eigenvalues of the matrix (26) have no positive real part, and the malware-free equilibrium *E*_0_ is locally asymptotically stable; when *R*_0_ > 1, Eq. (26) has a positive eigenvalue *α*_1_*N*_1_ − *γ* − *b*. Thus, malware-free equilibrium *E*_0_ is unstable. **Theorem 2.** The malware-free equilibrium *E*_0_ is globally asymptotically stable if *R*_0_ < 1.

**Proof.** Let

(26) \begin{eqnarray*}\begin{array}{@{}r@{}} \displaystyle D= \left\{ \begin{array}{@{}c@{}} \displaystyle (\widetilde {S},\widetilde {I},S,I)\in {R}^{4}\,\, \left\vert \,\,0\leqslant \widetilde {S}+\widetilde {I}\leqslant {N}_{1}, \right. \end{array} \right. \left. \begin{array}{@{}c@{}} \displaystyle 0\leqslant S+I\leqslant {N}_{2},\hspace*{10.00002pt}\widetilde {S},\widetilde {I},S,I\geqslant 0 \end{array} \right\} \end{array}\end{eqnarray*}

Obviously, *D* is the positive invariant set of system Eq. (13). When *R*_0_ < 1, construct the Liapunov function

(27) \begin{eqnarray*}V(t)=\widetilde {I}(t)\end{eqnarray*}

The derivative of *V*(*t*) along the trajectory of system Eq. (13). is

(28) \begin{eqnarray*}\begin{array}{@{}r@{}} \displaystyle \frac{dV \left( t \right) }{dt} = \frac{d\widetilde {I} \left( t \right) }{dt} & = \left[ {\alpha }_{1}\widetilde {S}- \left( b+\gamma \right) \right] \widetilde {I} & \\ \displaystyle & \leqslant \left[ {\alpha }_{1}{N}_{1}- \left( b+\gamma \right) \right] \widetilde {I} \end{array}\end{eqnarray*}

Since *R*_0_ < 1, then ${\alpha }_{1}{N}_{1}- \left( b+\gamma \right) \lt 0$. Thus,

(29) \begin{eqnarray*}Q= \left\{ \left( \widetilde {S},\widetilde {I},S,I \right) \in D \left\vert \frac{dV \left( t \right) }{dt} =0 \right. \right\} = \left\{ \widetilde {I}=0 \right\} \end{eqnarray*}

Therefore, the maximum invariant set of the system in *Q* is $ \left\{ \widetilde {I}=0 \right\} $. According to the principle of Lasalle invariance,

(30) \begin{eqnarray*}\lim _{t\rightarrow \infty }\widetilde {I} \left( t \right) =0\end{eqnarray*}

Substituting the preceding equation into system Eq. (13).

(31) \begin{eqnarray*}\lim _{t\rightarrow \infty }\widetilde {S} \left( t \right) ={N}_{1},\hspace*{10.00002pt}\lim _{t\rightarrow \infty }S \left( t \right) ={N}_{2},\hspace*{10.00002pt}\lim _{t\rightarrow \infty }I \left( t \right) =0\end{eqnarray*}

Therefore, *E*_0_ is globally attractive. Combined with the local stability of *E*_0_, it can be seen that *E*_0_ is globally asymptotically stable.

### Endemic equilibrium and its stability

**Lemma 3.** The endemic equilibrium *E*_1_ of system Eq. (13) is locally asymptotically stable if *R*_0_ > 1.

**Proof.** At the endemic equilibrium point, the Jacobian matrix is

(32) \begin{eqnarray*}J \left( {E}_{1} \right) = \left[ \begin{array}{@{}cccc@{}} \displaystyle -{\alpha }_{1}{\widetilde {I}}_{1}-b&\displaystyle -\gamma -b&\displaystyle 0&\displaystyle 0\\ \displaystyle {\alpha }_{1}{\widetilde {I}}_{1}&\displaystyle 0&\displaystyle 0&\displaystyle 0\\ \displaystyle 0&\displaystyle - \frac{b{N}_{1}}{{\widetilde {I}}_{1}+ \frac{b}{{\alpha }_{2}} } &\displaystyle -{\alpha }_{2}{\widetilde {I}}_{1}-b&\displaystyle 0\\ \displaystyle 0&\displaystyle \frac{b{N}_{1}}{{\widetilde {I}}_{1}+ \frac{b}{{\alpha }_{2}} } &\displaystyle {\alpha }_{2}{\widetilde {I}}_{1}&\displaystyle -\gamma -b \end{array} \right] \end{eqnarray*}

Two eigenvalues are given as

(33) \begin{eqnarray*}\lambda =-{\alpha }_{2}{\widetilde {I}}_{1}-b,\hspace*{10.00002pt}-\gamma -b\end{eqnarray*}

and are negative. The remaining two eigenvalues are given by

(34) \begin{eqnarray*}{\lambda }^{2}+({\alpha }_{1}{\widetilde {I}}_{1}+b)\lambda +(\gamma +b){\alpha }_{1}{\widetilde {I}}_{1}=0\end{eqnarray*}

According to the Routh–Hurwitz criteria, since all the coefficients of Eq. (36) are positive, there is no positive real part eigenvalue. When *R*_0_ > 1, all eigenvalues of the matrix Eq. (34) have no positive real part, and endemic equilibrium *E*_1_ is locally asymptotically stable.

**Theorem 4.** The endemic equilibrium *E*_1_ is globally asymptotically stable if *R*_0_ > 1.

**Proof.** When *R*_0_ > 1, construct the Liapunov function

(35) \begin{eqnarray*}V \left( t \right) = \frac{1}{2} {\omega }_{1}{ \left( \widetilde {S}-{\widetilde {S}}_{1} \right) }^{2}+{\omega }_{2} \left( \widetilde {I}-{\widetilde {I}}_{1}-{\widetilde {I}}_{1}\ln \nolimits \frac{\widetilde {I}}{{\widetilde {I}}_{1}} \right) \end{eqnarray*}

where *ω*_*i*_ > 0, i =1 , 2. The derivative of *V*(*t*) along the trajectory is

(36) \begin{eqnarray*}\begin{array}{@{}rl@{}} \displaystyle \frac{dV \left( t \right) }{dt} & ={\omega }_{1} \left( \widetilde {S}-{\widetilde {S}}_{1} \right) \frac{d\widetilde {S} \left( t \right) }{dt} +{\omega }_{2} \left( 1- \frac{{\widetilde {I}}_{1}}{\widetilde {I}} \right) \frac{d\widetilde {I} \left( t \right) }{dt} & \\ \displaystyle & ={\omega }_{1} \left( \widetilde {S}-{\widetilde {S}}_{1} \right) \left( b{N}_{1}-{\alpha }_{1}\widetilde {S}\widetilde {I}-b\widetilde {S} \right) +{\omega }_{2} \left( \widetilde {I}-{\widetilde {I}}_{1} \right) \left( {\alpha }_{1}\widetilde {S}-{\alpha }_{1}{\widetilde {S}}_{1} \right) &\displaystyle \\ \displaystyle & =-{\omega }_{1}{ \left( \widetilde {S}-{\widetilde {S}}_{1} \right) }^{2} \left( {\alpha }_{1}\widetilde {I}+b \right) +{\alpha }_{1} \left( {\omega }_{2}-{\omega }_{1}{\widetilde {S}}_{1} \right) \left( \widetilde {I}-{\widetilde {I}}_{1} \right) \left( \widetilde {S}-{\widetilde {S}}_{1} \right) &\displaystyle \\ \displaystyle \end{array}\end{eqnarray*}

Letting ${\omega }_{2}={\omega }_{1}{\widetilde {S}}_{1}$, and any value *ω*_1_ > 0, one thus obtains

(37) \begin{eqnarray*} \frac{dV \left( t \right) }{dt} =-{\omega }_{1}{ \left( \widetilde {S}-{\widetilde {S}}_{1} \right) }^{2} \left( {\alpha }_{1}\widetilde {I}+b \right) \leqslant 0\end{eqnarray*}

and, obviously,

(38) \begin{eqnarray*}Q= \left\{ \left( \widetilde {S},\widetilde {I},S,I \right) \in D \left\vert \frac{dV \left( t \right) }{dt} =0 \right. \right\} = \left\{ \widetilde {S}={\widetilde {S}}_{1} \right\} \end{eqnarray*}

where *D* is determined by Eq. (28). Therefore, the maximum invariant set of the system in *Q* is $ \left\{ \widetilde {S}={\widetilde {S}}_{1} \right\} $According to the principle of Lasalle invariance,

(39) \begin{eqnarray*}\lim _{t\rightarrow \infty }\widetilde {S} \left( t \right) ={\widetilde {S}}_{1}\end{eqnarray*}

Substituting this into system Eq. (13), one has

(40) \begin{eqnarray*}\lim _{t\rightarrow \infty }\widetilde {I} \left( t \right) ={\widetilde {I}}_{1},\hspace*{10.00002pt}\lim _{t\rightarrow \infty }S \left( t \right) ={S}_{1},\hspace*{10.00002pt}\lim _{t\rightarrow \infty }I \left( t \right) ={I}_{1}\end{eqnarray*}

Therefore, *E*_1_ is globally attractive. Combined with the local stability of *E*_1_, it can be seen that *E*_1_ is globally asymptotically stable.

## Simulation and Numerical Analysis

According to Theorem 2 and Theorem 4, the size of basic reproductive number *R*_0_ is of great significance in determining whether WSN malware will continue to propagate. When *R*_0_ < 1, the system reaches global stability at the malware-free equilibrium point regardless of the initial state of each group in the network, and the malware will eventually disappear. However, when *R*_0_ > 1, the system reaches global stability at the epidemic equilibrium point, and the number of infected nodes in the final system will be maintained at a normal state. Therefore, one can manipulate the size of the basic reproductive number *R*_0_ by changing network parameters, for example node density, communication radius, and death rate, to determine the effects of these parameters on malware propagation. To verify the results, a numerical simulation experiment was conducted using the system dynamics modeling software Vensim.

It is assumed that the number of cluster head nodes *N*_1_ = 100, and the number of common nodes *N*_2_ = 1000 in a WSN, and a set of simulation parameters was chosen as follows: *b* = 0.01, *γ* = 0.01, *σ* = 0.5, *r* = 1, *ɛ* = 8, *ν* = 1, *τ* = 5, *e* = 50, and *β* = 0.01. Initial values of susceptible, infected, and recovered nodes in WSNs are $\widetilde {S}(0)=75$, $\widetilde {I}(0)=25$, *S*(0) = 1000, *I*(0) = 0, and *R*(0) = 0. According to Eqs. (5) and (6), one can obtain the infection and recovery rates as 0.0061 and 0.0044 under the game of the malware and the defend system, respectively, and further calculate that *R*_0_ < 1.

**Figure 3:**
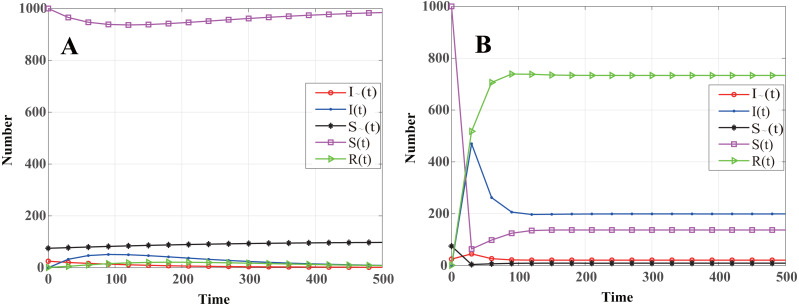
Dynamics showing that number of nodes in different states changes with time. (A) *R*_0_ < 1 (*β* = 0.0061, *γ* = 0.0044), the number of infected nodes is eventually reaches zero. (B) *R*_0_ > 1 (*β* = 0.3, *γ* = 0.0333), the number of infected nodes is eventually reaching a constant value at the disease equilibrium point *E*_1_.

The dynamics that the number of nodes in different states changes with time is shown in Fig. 3. Based on Fig. 3A, throughout the propagation of WSN malware, the number of infected cluster head nodes is monotonously decreasing, and eventually reaches zero; the number of infected common nodes increases rapidly in the initial stage, but as the number of cluster head infected nodes decreases, the number of common nodes infected gradually decreases, and eventually reaches zero. The other three state groups eventually reach a stable level. Therefore, the system state eventually reaches the malware-free equilibrium point *E*_0_, which is consistent with Theorem 2.

To further verify the propagation dynamics when *R*_0_ > 1, the loss detected by the system is reduced to *ν* = 5 and the revenue of node recovery increased to *e* = 10. At this time, the infection and recovery rates will increase to 0.3 and 0.0333, respectively. In this situation, one can obtain *R*_0_ > 1. The numerical simulation results are shown in Fig. 3B. It is found that the number of infected nodes increases rapidly in the initial stage, and then begins to decline before eventually reaching a constant value. Malware continues to propagate among network nodes, which shows the influence of the infection and recovery rates on malware propagation in WSNs. This is also in line with the conclusion obtained in Theorem 4, namely that the system is ultimately at the disease equilibrium point *E*_1_. Next, the effects of sensor-node density, node communication radius, and node death rate on WSN malware infection are studied.

### Node communication radius *r*

Letting *R*_0_ = 1 for Eq. (24), one can obtain the node communication radius threshold of the malware propagation in a cluster-based WSN:

(41) \begin{eqnarray*}{r}_{th}=\sqrt{ \left( \gamma +b \right) /\beta \sigma \pi }\end{eqnarray*}

**Figure 4:**
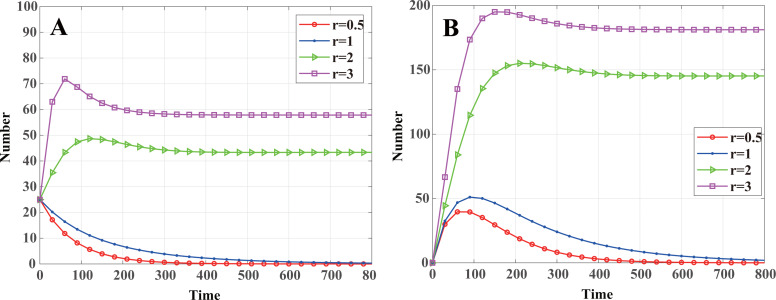
Dynamics of infected nodes for different communication radii *r*. (A) Infected cluster head nodes $\widetilde {I}(t)$, (B) Infected common nodes *I*(*t*). When *r* < *r*_*th*_, the malware in the system will eventually disappear; when *r* > *r*_*th*_, the number of infected nodes eventually tends to a constant value.

That is to say, when *r* < *r*_*th*_, *R*_0_ < 1, according to Theorem 2, system Eq. (13) will stabilize at the malware-free equilibrium *E*_0_, and the malware in the system will eventually disappear. When *r* > *r*_*th*_, *R*_0_ > 1, and, according to Theorem 4, system Eq. (13) will stabilize at the endemic equilibrium *E*_1_, and malware in WSNs will exist consistently. According to the aforementioned WSN parameters, one can calculate *r*_*th*_ = 1.2259. As shown in Fig. 4, when *r* = 0.5 < *r*_*th*_ and *r* = 1 < *r*_*th*_, system Eq. (13) stabilizes at malware-free equilibrium. The number of infected nodes eventually reaches zero, and convergence speed increases as *r* decreases. When *r* = 2 > *r*_*th*_ and *r* = 3 > *r*_*th*_, system Eq. (13) stabilizes at the endemic equilibrium. The number of infected nodes eventually tends to a constant value, and the constant value increases with *r*.

### Node distributed density *σ*

One can also obtain the node distributed density threshold of malware propagation in a cluster-based WSN according to Eq. (24):

(42) \begin{eqnarray*}{\sigma }_{th}= \frac{\gamma +b}{\beta \pi {r}^{2}} \end{eqnarray*}

**Figure 5:**
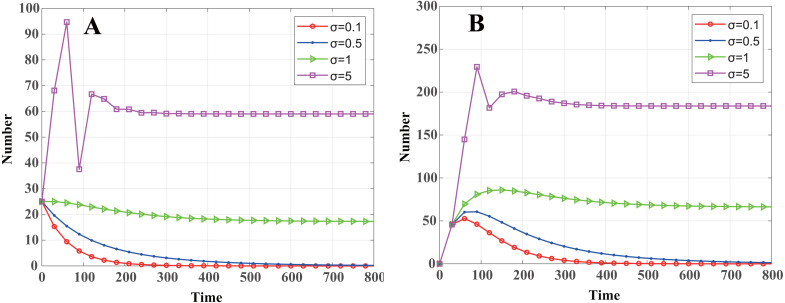
Dynamics of infected nodes for different distributed densities *σ*. (A) Infected cluster head nodes $\widetilde {I}(t)$, (B) Infected common nodes *I*(*t*). When *σ* < *σ*_*th*_, the malware in the system will eventually disappear; when *σ* > *σ*_*th*_, the number of infected nodes eventually tends to a constant value.

That is to say, when *σ* < *σ*_*th*_, *R*_0_ < 1, and the malware in the system will eventually disappear. When *σ* > *σ*_*th*_, *R*_0_ > 1, and system Eq. (13) will stabilize at the endemic equilibrium *E*_1_, and malware will exist consistently. By calculation, *σ* = 0.7514. As shown in Fig. 5, when *σ* = 0.1 < *σ*_*th*_ and *σ* = 0.5 < *σ*_*th*_, the malware will eventually disappear, and the convergence speed increases as *σ* decreases. When *σ* = 1 > *σ*_*th*_ and *σ* = 5 > *σ*_*th*_, the number of infected nodes eventually tends to a constant value, and the constant value increases with *σ*.

### Node death rate *b*

The threshold of malware propagation about the death rate of nodes is

(43) \begin{eqnarray*}{b}_{th}=\beta \sigma \pi {r}^{2}-\gamma \end{eqnarray*}

**Figure 6:**
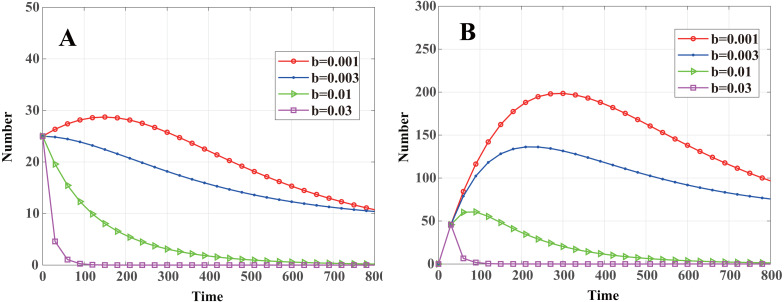
Dynamics of infected nodes for different death rates *b*. (A) Infected cluster head nodes $\widetilde {I}(t)$, (B) infected common nodes *I*(*t*). When *b* < *b*_*th*_, the number of infected nodes eventually tends to a constant value; when *b* > *b*_*th*_, the malware in the system will eventually disappear.

That is to say, when *b* > *b*_*th*_, *R*_0_ < 1, the malware in the system will eventually disappear. When *b* < *b*_*th*_, *R*_0_ > 1, system Eq. (13) will stabilize at the endemic equilibrium *E*_1_, and malware will exist consistently. By calculation, *b*_*th*_ = 0.0052. As shown in Fig. 6, when *b* = 0.001 < *b*_*th*_ and *b* = 0.003 < *b*_*th*_, system Eq. (13) stabilizes at endemic equilibrium. When *b* = 0.01 > *b*_*th*_ and *b* = 0.03 > *b*_*th*_, system Eq. (13) stabilizes at malware-free equilibrium. In contrast with communication radius *r* and node density *σ*, even when *R*_0_ is greater than 1, the number of infected cluster head nodes decreases monotonically and eventually tends to a constant value. In Figs. 4A and 5A, it can be seen that when *R*_0_ > 1 the number of infected cluster head nodes increases rapidly in the initial stage and then decreases to a constant level.

## Conclusion

In this paper, a differential equation model is proposed to analyze the propagation dynamic of malware based on epidemiology and game theory for cluster-based WSNs. The game between malware and the WSN defend system is established, and the model’s mixed strategy Nash equilibrium obtained. Different from the malware infection rate and recovery rate assumed in other existing propagation models, one can calculate the specific parameter expression of the infection and recovery rates according to the Nash equilibrium. When infectious disease theory is used to build a dynamic model of transmission, the communication methods of cluster head nodes and common nodes in a cluster-based network is considered and the malware propagation characteristics determined. The equilibrium point of the model is derived and the stability of the equilibrium point analyzed to determine conditions for avoiding the continuous propagation of malware in WSNs. The theoretical analysis and numerical simulations performed in this paper show that the propagation dynamics of malware in WSNs is closely related to node communication radius, node density, and node death rate. To effectively prevent and control the propagation of malicious software, cluster-based WSNs should set reasonable network parameters so that the basic reproductive number of malicious software propagation is less than 1, thereby improving WSN defense capabilities. In future work, we will further study the malware detection and defense issues in the WSN system, so that the system can detect malware in time and prevent damage.

##  Supplemental Information

10.7717/peerj-cs.728/supp-1 Supplemental Information 1 The Vensim numerical simulation file of the malware propagation model

## Additional Information and Declarations

### Data Availability

The following information was supplied regarding data availability: The Vensim numerical simulation file of the malware propagation model is available in the Supplementary File.
